# HLA-Cw*04 allele associated with nevirapine-induced rash in HIV-infected Thai patients

**DOI:** 10.1186/1742-6405-6-22

**Published:** 2009-10-21

**Authors:** Sirirat Likanonsakul, Tippawan Rattanatham, Siriluk Feangvad, Sumonmal Uttayamakul, Wisit Prasithsirikul, Preecha Tunthanathip, Emi E Nakayama, Tatsuo Shioda

**Affiliations:** 1 Bamrasnaradura Infectious Diseases Institute, Department of Disease Control, Ministry of Public Health, Nonthaburi, Thailand; 2 Research Institute for Microbial Diseases, Osaka University, Osaka, Japan

## Abstract

**Background:**

A high incidence of rash has been reported in HIV-1 patients who received the anti-retroviral drug nevirapine. In addition, several studies have suggested that polymorphisms of human leukocyte antigen (*HLA*) genes may play important roles in nevirapine-induced rash. The aim of the present study was to evaluate the effects of different *HLA-C *alleles on rash associated with nevirapine in patients who started highly active anti-retroviral therapy (HAART) containing nevirapine in Thailand.

**Results:**

A case-control study was carried out involving HIV-1 patients under treatment at Bamrasnaradura Infectious Diseases Institute, Nonthaburi, Thailand between March 2007 and March 2008. The study included all HIV/AIDS patients being treated with nevirapine-containing regimens. The study population comprised 287 HIV/AIDS patients of whom 248 were nevirapine-tolerant and 39 developed rash after nevirapine treatment. From the nevirapine-tolerant patients, 60 were selected as the control group on the basis of age, sex, and therapy history matched for nevirapine-induced rash cases. We observed significantly more HLA-Cw*04 alleles in nevirapine-induced rash cases than in nevirapine-tolerant group, with frequencies of 20.51% and 7.50%, respectively (P = 0.009). There were no significant differences between the rash and tolerant groups for other *HLA-C *alleles except for HLA-Cw*03 (P = 0.015).

**Conclusion:**

This study suggests that HLA-Cw*04 is associated with rash in nevirapine treated Thais. Future screening of patients' *HLA *may reduce the number of nevirapine-induced rash cases, and patients with alleles associated with nevirapine-induced rash should be started on anti-retroviral therapy without nevirapine.

## Background

Highly active antiretroviral therapy (HAART) has significantly improved the prognosis of HIV-1-infected patients and prolonged AIDS-free survival[1]. HAART has also resulted in immune restoration and reduction of morbidity and mortality even for patients with advanced HIV-1 infection[1,2]. Nevirapine (NVP) is a non-nucleoside reverse transcriptase inhibitor (NNRTI) that has been shown to have high antiretroviral efficacy [3]. NVP-based HAART regimens have therefore been widely used in resource-limited countries because of their efficacy, availability and relatively low cost. In Thailand, the Government Pharmaceutical Organization (GPO) has produced GPOvir, a low cost (US$ 30 per month) and fixed-dose combination of NVP, stavudine (D4T), and lamivudine (3TC), which has been commercially available since March 2002.

However, NVP-associated rash has been reported to be as high as 48% after the start of treatment with this inhibitor [4]. Nearly 90% of the side effects of GPOvir are thought to be due to NVP hypersensitivity [5]. Skin rash is the most common adverse drug reaction associated with NVP, and hypersensitivity reaction to NVP is rapid and severe when drug administration is suspended and re-challenged [6]. Most patients develop rash between the first and third week of treatment [7], including the more severe forms of rash such as extensive maculopapular rash, serum sickness-like reaction, hypersensitivity syndrome, Steven-Johnson syndrome and toxic epidermal necrosis [7,8]. NVP-induced rash has been reported in 4.3-36% of adults [9,10] with the incidence for Thai patients ranging from 6 to 21% [5], reflecting the comparatively high incidence of rash in Asians [11]. Several features of NVP hypersensitivity suggest that genetic factors may play an important predisposing role in NVP hypersensitivity, in which NVP itself or NVP-induced antigens may trigger an immunological response that is dependent on CD4 T lymphocytes in susceptible hosts. This supports the hypothesis that the hypersensitivity reaction to NVP may be HLA-associated [9,12,13], while HLA-alleles have also been identified as clinically relevant susceptibility markers for hypersensitivity reaction to another antiretroviral drug [14]. Recent studies have shown that in Japan the HLA-Cw*08 allele is associated with NVP hypersensitivity [15]. The objective of the study presented here was to compare allele frequency of *HLA-C *in Thai patients with rash who had to change from GPOvir to a regime containing efavirenz and those who were NVP-tolerant.

## Results

A case-control study was carried out. The study population comprised 287 HIV/AIDS patients of whom 248 were nevirapine-tolerant and 39 developed rash. From the nevirapine-tolerant patients, 60 were selected as the control group on the basis of age, sex, and therapy history matched for nevirapine-induced rash cases. As shown in Table 1, the demographic and clinical characteristics of patients with NVP-induced rash were very similar to those of NVP-tolerant patients. The medians of CD4 cell counts of NVP-induced rash and NVP-tolerant group were comparable at both time points of immediately before NVP treatment and 6 month after treatment. It is known that HIV-infected patients frequently suffered from allergic drug reactions [16]. Nearly all of rash cases (37 out of 39) used steroids and more than half (35 out of 60 cases) of NVP-tolerant patients also used steroids. Four out of 35 NVP-tolerant steroid users had developed mild rash that could be controlled by steroid. Remaining 31 NVP-tolerant patients used steroid to suppress allergic reactions including chronic allergic skin, rhinitis, asthma, and drug reactions upon Pneumocystis carinii pneumonia treatment, and immune reconstitution inflammatory syndrome. The NVP-induced rash cases manifested severe rash, which could not be suppressed by steroid, and had to change the regimen.

**Table 1:** Demographics and immunological variables of the NVP-induced rash and NVP-tolerant groups

**Variables**	**NVP-induced rash**	**NVP-tolerant**	**P value**
	**(n = 39)**	**(n = 60)**	
Age (median IQR)	39.0 (34.0-44.0)	38.0 (35.0-41.75)	*0.71
			
Sex [n(%)]			**0.68
Male	22 (56.41%)	31 (51.67%)	
Female	17 (43.59%)	29 (48.33%)	
			
Duration of Treatment, year (median IQR)	1 (0-3)	1 (0-2.75)	*0.47
			
Pre-NVP-treatment CD4 T-cell count × 10^6^/l	43.5	55	*0.71
(median IQR)	(19.50-135.50)	(28.25-137.50)	
			
Post-NVP-treatment CD4 T-cell count × 10^6^/l	243	241	*0.98
(median IQR)	(155.00-328.00)	(185.00-312.25)	

*Mann-Whitney U-test

**Chi square test

The frequencies of the *HLA-C *alleles identified in the 39 samples in the NVP-induced rash group and 60 samples in the NVP tolerant group are presented in Table 2. Frequency of HLA-Cw*04 was approximately 21% for the patients with NVP-induced rash and 7.5% for the NVP-tolerant group, showing a statistically significant difference in HLA-Cw*04 allele frequency (P = 0.0088, Fisher's exact test). The reported HLA-Cw*04 allele frequency for the normal Thai population (0.102) [17] is higher than that of the NVP-tolerant group (0.075) and lower than that of the NVP-induced rash group (0.205). Although statistical significance of this difference was lost after stringent Bonferroni correction (Pc = 0.088), these results suggested that HLA-Cw*04 was associated with NVP-induced rash in HIV-1 infected Thai patients. One-third of the patients with NVP-induced rash (13 out of 39) carried HLA-Cw*04 in comparison with 15% of the NVP-tolerant patients (9 out of 60) (P = 0.047, Fisher's exact test; Pc = 0.47). When we compared 39 NVP-induced rash patients with 25 NVP-tolerant patients who did not receive steroid, the concentration of HLA-Cw*04 allele in NVP rash cases was still apparent (0.205 vs 0.060, P = 0.04, Fisher's exact test).

**Table 2:** Occurrence of HLA-C alleles in the nevirapine (NVP)-rash cases and NVP-tolerant controls in Thailand

**Allele**	**NVP-induced rash number (%)**	**NVP-tolerant number (%)**	*P value
HLA-Cw*01	12 (15.38)	12 (10.00)	NS
HLA-Cw*03	4 (5.13)	20 (16.67)	0.01
HLA-Cw*04	16 (20.51)	9 (7.50)	0.009
HLA-Cw*05	2 (2.56)	3 (2.50)	NS
HLA-Cw*06	8 (10.26)	9 (7.50)	NS
HLA-Cw*07	19 (24.36)	39 (35.50)	NS
HLA-Cw*08	9 (11.54)	15 (12.50)	NS
HLA-Cw*12	6 (7.69)	8 (6.67)	NS
HLA-Cw*14	0	3 (2.50)	NS
HLA-Cw*15	2 (2.56)	2 (1.67)	NS

Total	78	120	

*Fisher's exact test

NS: P > 0.05

In contrast to HLA-Cw*04, fewer HLA-Cw*03 alleles were found in patients with NVP-induced rash than in NVP-tolerant ones (0.051 and 0.167) (P = 0.015, Fisher's exact test; Pc = 0.15). Approximately 7.7% of patients with NVP-induced rash (3 out of 39) carried HLA-Cw*03 in comparison with 30% of the NVP-tolerant patients (18 out of 60) (P = 0.011 Fisher's exact test, Pc = 0.11). There were no significant differences between the NVP-induced rash and NVP-tolerant groups in allele frequencies of HLA-Cw*01, HLA-Cw*05, HLA-Cw*06, HLA-Cw*07, HLA-Cw*08, HLA-Cw*12, HLA-Cw*14, and HLA-Cw*15. Other HLA-Cw* alleles were not detected in the tested samples. The NVP-tolerant group allele frequencies of HLA-Cw*03 (0.167), HLA-Cw*08 (0.125) and HLA-Cw*12 (0.067) were very similar to those of the normal Thai population (0.174, 0.144 and 0.06, respectively) [17], which suggests that our genotyping was accurate.

HLA-DRB1*01 was reported to be associated with NVP hypersensitivity in Australian [13] and French [18] cohorts. We therefore performed PCR-sequence specific oligonucleotide probe (SSOP) method to detect HLA-DRB1*01 alleles. Contrary to our expectation, we failed to detect HLA-DRB1*01 allele in 39 NVP-induced rash cases, while we detected five of this allele in 60 NVP-tolerant patients. This result suggested that the HLA-DRB1*01 allele may not involved in NVP rash in Thai population.

The C allele of the SNP rs9264942, located in the 5'upstream region of the *HLA-C *gene, was reported to associate with higher levels of HLA-C expression and HLA-Cw*04 allele [19]. To know whether or not higher levels of *HLA-C *gene expression associated with NVP-induced rash, we genotyped rs9264942 SNP by TaqMan SNP genotyping system. The C allele frequency in 39 NVP rash cases was 0.45, while it was 0.38 in 60 NVP-tolerant patients (P = 0.36, Chi square test). This result suggested that the HLA-Cw*04 allele itself rather than the relative high levels of HLA-C expression was involved in NVP-induced rash.

## Discussion

In the study reported here, we genotyped *HLA-C *alleles of 39 patients with NVP-induced rash and 60 NVP-tolerant Thai patients, and found that frequency of HLA-Cw*04 was higher in NVP-induced rash Thai patients than in NVP-tolerant patients. While the number of samples in our study is small, the increased frequency of HLA-Cw*04 in patients with NVP-induced rash suggested that this allele plays an important role in the development of rash after GPOvir treatment. HLA-Cw*04 was found to be associated with rapid development of AIDS-defining conditions in Caucasians [20,21] but to have a protective effect in African Americans [22]. In hepatitis C virus infection cases, HLA-Cw*04 was associated with viral persistence [23].

Previous studies [15,24] have suggested that HLA-Cw*08 is associated with NVP hypersensitivity. Littera *et al*. studied 49 Sardinian HIV-1-positive patients treated with NVP and reported that HLA-Cw*08 and/or HLA-B*14(65) is associated with NVP hypersensitivity [24]. Subsequently, Gatanaga *et al*. studied HIV-1 infected individuals in Japan [15]. In this study, 41 patients had a history of NVP treatment, 12 of whom showed NVP hypersensitivity. The frequency of HLA-B*14 is nearly 0% in Japan. On the other hand, the frequency of HLA-Cw*08-positive patients in the NVP hypersensitive group was 42%, which was significantly higher than that the NVP tolerant group (10%) [15]. In our study, however, the frequencies of HLA-Cw*08 in the NVP rash and tolerant groups were 0.115 and 0.125, respectively, without any significant difference between the two groups (P = 1.000, Fisher's test). Although the precise reason for the difference between the findings of these previous studies and ours is not clear at present, there were several differences between them. First, we genotyped approximately three times more patients with NVP-induced rash than was done in the previous studies. Second, our study focused on rash after NVP treatment but the other studies dealt with patients with rash and/or hepatotoxicity. Furthermore, it is possible that the levels of linkage disequilibrium between *HLA-C *alleles and those of other HLA locus and/or other genes differ among different ethnic groups. As described above, HLA-Cw*04 was associated with rapid AIDS progression in Caucasians [20,21] but to have a protective effect in African Americans [22]. Thus, it is possible that other SNP(s) or HLA allele(s) responsible for NVP-induced rash is in a stronger linkage disequilibrium with HLA-Cw*04 in Thais than the other ethnic groups tested previously.

Previous studies have also suggested that higher CD4 counts at baseline increased risk of NVP-induced rash [25-30]. In our study, however, the median baseline CD4 counts of NVP-induced rash cases (43.5 cells/μl) was very similar to that of 248 NVP-tolerant patients (43 cells/μl). The effects of high baseline CD4 counts on risk of NVP-induced rash were reportedly observed mainly in patients whose CD4 counts were over 250 cells/μl [29]. Accordingly, nearly all of patients in our study started NVP-containing regimen after their CD4 counts dropped below 250 cells/μl. Therefore, it is also possible that low baseline CD4 counts in our study limit the ability to detect an HLA association reported previously [15,24]. Nevertheless, our results are practically meaningful since most of HIV-1-infected individuals in Thailand start antiretroviral treatment after their CD4 counts drop below 250 cells/μl.

One report from Thailand demonstrated that the effects of high baseline CD4 counts on risk of NVP-induced rash were still observed even in patients whose baseline CD4 counts were below 250 cells/μl, although the levels of such effects was very small in patients whose baseline CD4 counts were below 200 cells/μl [30]. Therefore, we divided patients according to their baseline CD4 counts. When we picked up patients whose baseline CD4 counts were over 100 cells/μl, HLA-Cw*04 allele frequency was 0.25 in 14 NVP-induced rash cases and 0.025 in 20 NVP-tolerant patients. Statistical significance of this difference rather increased in these groups (P = 0.0068). When we picked up patients whose baseline CD4 were less than 100 cells/μl, HLA-Cw*04 allele frequency was 0.2045 in 22 NVP-induced rash cases and 0.0897 in 39 NVP-tolerant patients. Although there was an apparent trend towards high HLA-Cw*04 frequency in NVP-induced rash cases, statistical significance of the difference greatly reduced in these groups (P = 0.094). Therefore, the difference in the allele frequency was more prominent in patients with higher CD4 counts even in our patient group.

During the preparation of this manuscript, Chantarangsu *et al*. reported a strong association between HLA-B*3505 and NVP-induced skins rash in HIV-infected Thai patients [31]. Our results at least partly confirm theirs since HLA-Cw*0401 showed the second highest levels of difference in allele frequency between NVP-rash and NVP-tolerant patients in their study [31]. It is known that HLA-Cw*04 is in a linkage disequilibrium with HLA-B*35 in Thais [32]. It is, thus, necessary to investigate whether or not HLA-B*3505 is also overrepresented in NVP-rash cases in our study.

## Conclusion

We observed higher frequency of HLA-Cw*04 in NVP-induced skin rash than in NVP-tolerant patients in Thailand. In addition to HLA-Cw*04 and/or HLA-B*3505, future screening of patients' *HLA *and genes involved in hypersensitive reactions may identify other alleles responsible for the incidence of NVP-induced rash. Patients possessing alleles responsible for NVP-rash should be started on anti-retroviral therapy without NVP.

## Methods

### Clinical specimens

A case-control study was carried out with HIV-1 infected patients who were under treatment at Bamrasnaradura Infectious Diseases Institute, Ministry of Public Health, Nonthaburi, Thailand. The targeted study population comprised 672 HIV-1/AIDS patients and the study period ran from March 2007 to March 2008. Patients who developed apparent skin rash anywhere on the body after NVP containing HAART and had to change their NVP-containing regime to efavirenz-containing ones were diagnosed as rash. There were 39 patients who matched these criteria. Most of these 39 patients developed rash within two months after NVP treatment (NVP-rash), and none of them showed liver toxicity. On the other hand, 248 patients showed reasonably good adherence to NVP and did not develop rash at all or developed only mild rash that could be controlled by steroid within the observation period. The remaining 385 patients were excluded because of treatment without NVP (184 cases), incomplete clinical records (101 cases), treatment interruptions (62 cases), adverse drug effects other than rash (18 cases), and incomplete HIV-1 suppression (20 cases). From the 248 NVP tolerant patients, we first tried to have two control patients for each rash case matching age, sex, and duration of therapy before NVP containing HAART. However, some rash cases have only one control mainly due to the limitation of available reagents. Total of 60 samples were thus selected for the control group (NVP-tolerant). Age, sex, treatment history and CD4 cell counts were not different between test and control groups as shown in Table 1. Two hundred μl of whole blood was collected from each of those patients and kept at -20°C until DNA extraction with the QIAamp DNA Blood Mini Kit (QIAGEN, Hilden, Germany). All participants signed informed consent forms. The present study was approved by the institutional ethics committees of the Bamrasnaradura Infectious Diseases Institute and the Department of Diseases Control, Ministry of Public Health, Thailand.

### HLA-C Typing

Medium-high resolution HLA-C typing was performed with an HLA-C typing kit (MPH-2 HLA-C typing kit, Wakunaga, Japan) according to the manufacturer's instructions. Any ambiguous results were checked by nucleotide sequence determination of PCR-amplified DNA fragments of HLA-C exons 2, and 3 [33]. GeneAmp^® ^PCR system 9600 (Applied Biosystems, Foster City. CA) was used for all the PCR reactions and DNA sequencer 373 (Applied Biosystems) was used for determination of the nucleotide sequence of an amplified fragment.

### HLA-DRB1*01 detection

We performed HLA class II DNA-based typing of DR1 as described in the 12th International Histocompatibility Working Group version 1.5. Amplified DNA with primer pair 2DRBAMP1 (5'-TTCTTGTGGCAGCTTAAGTT-3') and 2DRBAMP-B(5'-CCGCTGCACTGTGAAGCTCT-3') in exon 2 was treated with NaOH and the denatured DNA was loaded onto a nylon membrane manually using a milliblot system with a vacuum manifold. UV light was used to crosslink the DNA to the membrane. For hybridization with DR1 specific probe, we used DRB 1001w (5'-TGGCAGCTTAAGTTTGAA-3') digoxigenin-labeled SSOP for detection of HLA-DRB1*01 positive sample. After stringent wash procedure, the membrane was incubated with an antibody to digoxigenin coupled with alkaline phosphatase. Addition of a substrate for alkaline phosphatase caused light to be emitted by the Lumiphos. This light was detected by exposure of X-ray film.

### HLA-C 5' SNP genotyping

The rs9264942 SNP genotyping was performed by TaqMan SNP genotyping system with ABI real time PCR 7300. A validated primer and probe mix (C__29901957_10) were purchased from Applied Biosystems.

### Statistical analysis

Differences in age, duration of therapy before start of NVP containing HAART, and pre- and post-therapy CD4 cell counts between case and control groups were evaluated by Mann-Whitney U test. A difference in proportion of sex was evaluated by Chi square test. Differences in the allele frequencies between the two groups were evaluated by Fisher's exact test. P values less than 0.05 were considered to be statistically significant. The corrected P (Pc) values were calculated by using Bonferroni's correction.

## Competing interests

The authors declare that they have no competing interests.

## Authors' contributions

SL conceived of the study, participated in the design and coordination of the study, and drafted the manuscript, TR carried out genotyping and analysis of clinical data, SF carried out genotyping and analysis of clinical data, SU participated in coordination of the study and helped to draft the manuscript. WP participated in collection of clinical data and helped to draft the manuscript, PT participated in coordination of the study and helped to draft the manuscript, EEN supervised genotyping, participated in study design and helped to draft the manuscript, TS participated in the design of the study, performed the statistical analysis, and helped to draft the manuscript. All authors read and approved the final manuscript.

## Author's information

SL is a chief of Immunology and Virology Laboratory, Bamrasnaradura Infectious Diseases Institute, which is a governmental institute with the largest infectious disease hospital in Thailand. TR and SF are research assistants of the study. SU is a sub-chief of Immunology and Virology Laboratory and working on HIV-1 diagnosis. WP is a clinician who is taking care of HIV-1 infected patients. PT is a director of Bamrasnaradura Infectious Diseases Institute. EEN is an assistant professor of Osaka University, Japan. TS is a professor of Osaka University working on HIV-1 infection and host genome.
